# Supplementary material for: Identification of photoperiod-induced specific miRNAs in the adrenal glands of Sunite sheep (Ovis aries)
Source: Front Vet Sci. 2022 Jul 22;9:888207. doi: 10.3389/fvets.2022.888207 (PMC9354845; doi:10.3389/fvets.2022.888207)
Supplement: Supplementary file 1 [file Data_Sheet_1.ZIP › Supplementary file/miRDeep2 file.zip!/miRDeep2 file/Know_Structure/LP42A3/oar-mir-22.pdf]

miRBase precursor : oar-mir-22
Total read count : 72790
oar-miR-22-3p read count : 67978
remaining reads : 4812

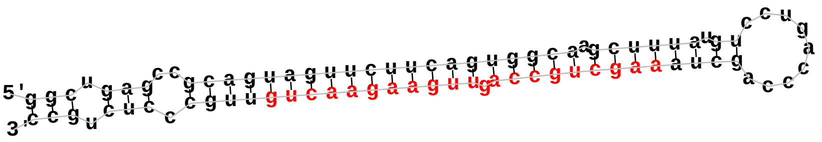

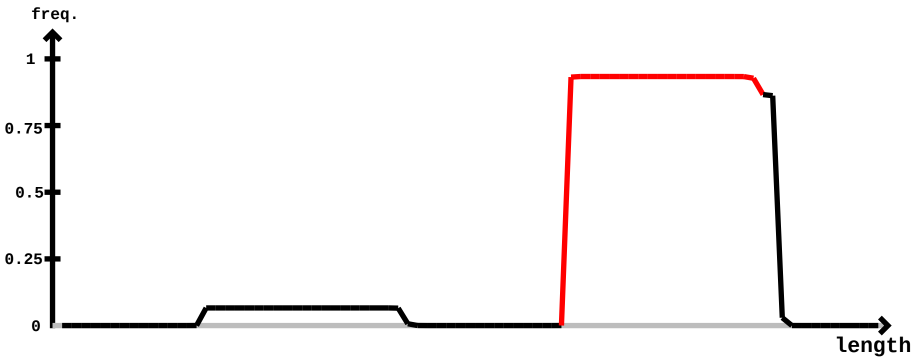

oar-miR-22-3p

```
5'- ggcugagccgcaguaguucuucaguggcaagcuuuauguccugacccagcuaaagcugccaguugaagaacuguugcccucugcc  -3'    exp
    (((.(((..((((((((((((((((((.((((((.((.........)))))))))))))).))))))))))))))).))).)))    reads      mm          sample
    .............uaguucuucaguggcaagcu....................................................    1          0           seq
    .............uaguucuucaguggcaagcuuu..................................................    10         0           seq
    ..............aguucuucaguggcaagc.....................................................    1          0           seq
    ..............aguucuucaguggcaagU.....................................................    1          1           seq
    ..............aguucuucaguggcaagcu....................................................    1          0           seq
    ..............aguucuucaguggcaagUuu...................................................    1          1           seq
    ..............aguucuucaguggcaagcuu...................................................    51         0           seq
    ..............aguucuucaguggcaagcuuu..................................................    4247       0           seq
    ..............aguucuucaguggcaagcuuua.................................................    475        0           seq
    ..............aguucuucaguggcaagcuuuau................................................    20         0           seq
    ...............guucuucaguggcaagcuuu..................................................    3          0           seq
    ...............guucuucaguggcaagcuuua.................................................    1          0           seq
    ...................................................aaagcugccaguugaagaacu.............    5          0           seq
    ...................................................aaagcugccaguugaagaacugu...........    10         0           seq
    ....................................................aagcugccaguugaaga................    9          0           seq
    ....................................................aagcugccaguugaagaa...............    15         0           seq
    ....................................................aagcugccaguugaagaac..............    448        0           seq
    ....................................................aagcugccaguugaagaacu.............    4444       0           seq
    ....................................................aagcugccaguugaagaacug............    301        0           seq
    ....................................................aagcugccaguugaagaacugu...........    60457      0           seq
    ....................................................aagcugccaguugaagaacuguu..........    2129       0           seq
    ....................................................aagcugccaguugaagaacuguug.........    4          0           seq
    .....................................................agcugccaguugaagaacu.............    10         0           seq
    .....................................................agcugccaguugaagaacugu...........    134        0           seq
    .....................................................agcugccaguugaagaacuguu..........    11         0           seq
    ........................................................ugccaguugaagaacugu...........    1          0           seq
```
